# Supplementary material for: Extracurricular Functions of tRNA Modifications in Microorganisms
Source: Genes (Basel). 2020 Aug 7;11(8):907. doi: 10.3390/genes11080907 (PMC7466049; doi:10.3390/genes11080907)
Supplement: Supplementary file 1 [file genes-11-00907-s001.pdf]

Supplementary Information

Extracurricular Functions of tRNA Modifications in Microorganisms

Ashley M. Edwards, Maame A. Addo, Patricia C. Dos Santos*

Table S1. tRNA modifications with corresponding positions and varying types of stressors that affect each respective modification level

tRNA Modifications Affected by Nutritional and Environmental Stressors			
**Position**	**Modification**	**Type of Stressor**	**References**
34	mcm^5s^2U	Nutrient limitation	[1-3]
34	mnm5s2U	UVR, Oxidative stress, Cold stress	[4],[5-6],[7]
34	$cmnm^5s^2U$	UVR, Antibiotic/Virulence	[4], [8-10]
37	ms^2i^6A	Nutrient limitation, Oxidative stress, Hypoxia, UVR, Antibiotic/Virulence	[11-15], [6,16], [17], [4], [18-19]
34	m^5C	Oxidative stress	[20-21]
32	Cm	Oxidative stress	[20-22]
32	Am	Oxidative stress	[22]
32	Um	Oxidative stress	[22]
32	s^2C	Oxidative stress	[6,23]
26	$m^2{}_2G$	Oxidative stress	[20-21]
34	oQ	Hypoxia	[17]
58	m^1A	Heat stress	[24-25]
54	s^2T	Heat stress, Oxidative stress	[24,26-29], [5]
46	m^7G	Oxidative stress, Heat stress	[30,32], [31]
39 & 55	ψ	Heat stress, Cold stress	[33-34], [35]
34	ac^4Cm	Heat stress	[26]
15	G^+	Heat stress	[25]
26	$m^2{}_2Gm$	Heat stress	[26]
26	m^2Gm	Heat stress	[28]
57	m^1I	Heat stress	[25]
14-21	D	Cold stress	[36-37]
37	ms^2io^6A	Oxidative stress, Hypoxia	[16], [17]
34	xmo^5U	Hypoxia	[38]
34	Q	Nutrient limitation, Antibiotic/Virulence	[39-40], [19]

37	m^1G	Antibiotic/Virulence	[41]
8	s^4U	Oxidative stress, UVR	[5-6,42-46], [4,47-49]
34	s^2U	Oxidative stress	[50]

References

1. Laxman, S.; Sutter, B.M.; Wu, X.; Kumar, S.; Guo, X.; Trudgian, D.C.; Mirzaei, H.; Tu, B.P. Sulfur Amino Acids Regulate Translational Capacity and Metabolic Homeostasis through Modulation of tRNA Thiolation. *Cell* **2013**, *154*, 416–429, doi:10.1016/j.cell.2013.06.043.
2. Gupta, R.; Laxman, S. tRNA wobble-uridine modifications as amino acid sensors and regulators of cellular metabolic state. *Curr Genet* **2019**, doi:10.1007/s00294-019-01045-y.
3. Gupta, R.; Walvekar, A.S.; Liang, S.; Rashida, Z.; Shah, P.; Laxman, S. A tRNA modification balances carbon and nitrogen metabolism by regulating phosphate homeostasis. *Elife* **2019**, *8*, doi:10.7554/eLife.44795.
4. Sun, C.; Jora, M.; Solivio, B.; Limbach, P.A.; Addepalli, B. The Effects of Ultraviolet Radiation on Nucleoside Modifications in RNA. *ACS Chemical Biology* **2018**, *13*, 567–572, doi:10.1021/acschembio.7b00898.
5. Watanabe, K. Reactions of 2-thioribothymidine and 4-thiouridine with hydrogen peroxide in transfer ribonucleic acids from Thermus thermophilus and Escherichia coli as studied by circular dichroism. *Biochemistry* **1980**, *19*, 5542–5549, doi:10.1021/bi00565a013.
6. Rao, Y.S.P.; Cherayil, J.D. Studies on chemical modification of thionucleosides in the transfer ribonucleic acid of Escherichia coli. *Biochem J* **1974**, *143*, 285–294.
7. Singh, A.K.; Shivaji, S. A cold-active heat-labile t-RNA modification GTPase from a psychrophilic bacterium Pseudomonas syringae (Lz4W). *Research in Microbiology* **2010**, *161*, 46–50, doi:10.1016/j.resmic.2009.11.002.
8. Shippy, D.C.; Eakley, N.M.; Lauhon, C.T.; Bochsler, P.N.; Fadl, A.A. Virulence characteristics of Salmonella following deletion of genes encoding the tRNA modification enzymes GidA and MnmE. *Microb. Pathog.* **2013**, *57*, 1–9, doi:10.1016/j.micpath.2013.01.004.
9. Cho, K.H.; Caparon, M.G. tRNA modification by GidA/MnmE is necessary for Streptococcus pyogenes virulence: a new strategy to make live attenuated strains. *Infect. Immun.* **2008**, *76*, 3176–3186, doi:10.1128/IAI.01721-07.
10. Shippy, D.C.; Fadl, A.A. Immunological characterization of a gidA mutant strain of Salmonella for potential use in a live-attenuated vaccine. *BMC Microbiology* **2012**, *12*, 286, doi:10.1186/1471-2180-12-286.
11. Rhaese, H.-J.; Grade, R.; Dichtelmüller, H. Studies on the Control of Development. *European Journal of Biochemistry* **1976**, *64*, 205–213, doi:10.1111/j.1432-1033.1976.tb10289.x.
12. Menichi, B.; Heyman', T. Thiomethylation of Tyrosine Transfer Ribonucleic Acid Is Associated with Initiation of Sporulation in Bacillus subtilis: Effect of Phosphate Concentration. *J. BACTERIOL.* 4.
13. Buck, M.; Griffiths, E. Iron mediated methylthiolation of tRNA as a regulator of operon expression in Escherichia coli. *Nucleic Acids Res.* **1982**, *10*, 2609–2624, doi:10.1093/nar/10.8.2609.
14. Vecerek, B.; Moll, I.; Bläsi, U. Control of Fur synthesis by the non-coding RNA RyhB and iron-responsive decoding. *EMBO J.* **2007**, *26*, 965–975, doi:10.1038/sj.emboj.7601553.
15. Pollo-Oliveira, L.; de Crécy-Lagard, V. Can protein expression be regulated by modulation of tRNA modifications profiles? *Biochemistry* **2019**, *58*, 355–362, doi:10.1021/acs.biochem.8b01035.
16. Skovran, E.; Lauhon, C.T.; Downs, D.M. Lack of YggX Results in Chronic Oxidative Stress and Uncovers Subtle Defects in Fe-S Cluster Metabolism in Salmonella enterica. *Journal of Bacteriology* **2004**, *186*, 7626–7634, doi:10.1128/JB.186.22.7626-7634.2004.

17. Buck, M.; Ames, B.N. A modified nucleotide in tRNA as a possible regulator of aerobiosis: synthesis of cis-2-methyl-thioribosylzeatin in the tRNA of Salmonella. *Cell* **1984**, *36*, 523–531, doi:10.1016/0092-8674(84)90245-9.
18. Durand, J.M.; Björk, G.R.; Kuwae, A.; Yoshikawa, M.; Sasakawa, C. The modified nucleoside 2-methylthio-N6-isopentenyladenosine in tRNA of Shigella flexneri is required for expression of virulence genes. *J. Bacteriol.* **1997**, *179*, 5777–5782, doi:10.1128/jb.179.18.5777-5782.1997.
19. Durand, J.M.B.; Dagberg, B.; Uhlin, B.E.; Björk, G.R. Transfer RNA modification, temperature and DNA superhelicity have a common target in the regulatory network of the virulence of Shigella flexneri: the expression of the virF gene. *Molecular Microbiology* **2000**, *35*, 924–935, doi:10.1046/j.1365-2958.2000.01767.x.
20. Chan, C.T.Y.; Pang, Y.L.J.; Deng, W.; Babu, I.R.; Dyavaiah, M.; Begley, T.J.; Dedon, P.C. Reprogramming of tRNA modifications controls the oxidative stress response by codon-biased translation of proteins. *Nat Commun* **2012**, *3*, 937, doi:10.1038/ncomms1938.
21. Chan, C.T.Y.; Dyavaiah, M.; DeMott, M.S.; Taghizadeh, K.; Dedon, P.C.; Begley, T.J. A Quantitative Systems Approach Reveals Dynamic Control of tRNA Modifications during Cellular Stress. *PLOS Genetics* **2010**, *6*, e1001247, doi:10.1371/journal.pgen.1001247.
22. Jaroensuk, J.; Atichartpongkul, S.; Chionh, Y.H.; Wong, Y.H.; Liew, C.W.; McBee, M.E.; Thongdee, N.; Prestwich, E.G.; DeMott, M.S.; Mongkolsuk, S.; et al. Methylation at position 32 of tRNA catalyzed by TrmJ alters oxidative stress response in Pseudomonas aeruginosa. *Nucleic Acids Res* **2016**, *44*, 10834–10848, doi:10.1093/nar/gkw870.
23. Romsang, A.; Duang-nkern, J.; Khemsom, K.; Wongsaroj, L.; Saninjuk, K.; Fuangthong, M.; Vattanaviboon, P.; Mongkolsuk, S. Pseudomonas aeruginosa ttcA encoding tRNA-thiolating protein requires an iron-sulfur cluster to participate in hydrogen peroxide-mediated stress protection and pathogenicity. *Scientific Reports* **2018**, *8*, doi:10.1038/s41598-018-30368-y.
24. Shigi, N.; Suzuki, T.; Terada, T.; Shirouzu, M.; Yokoyama, S.; Watanabe, K. Temperature-dependent biosynthesis of 2-thioribothymidine of Thermus thermophilus tRNA. *J. Biol. Chem.* **2006**, *281*, 2104–2113, doi:10.1074/jbc.M510771200.
25. Orita, I.; Futatsuishi, R.; Adachi, K.; Ohira, T.; Kaneko, A.; Minowa, K.; Suzuki, M.; Tamura, T.; Nakamura, S.; Imanaka, T.; et al. Random mutagenesis of a hyperthermophilic archaeon identified tRNA modifications associated with cellular hyperthermotolerance. *Nucleic Acids Res* **2019**, *47*, 1964–1976, doi:10.1093/nar/gky1313.
26. Kowalak, J.A.; Dalluge, J.J.; McCloskey, J.A.; Stetter, K.O. The Role of Posttranscriptional Modification in Stabilization of Transfer RNA from Hyperthermophiles. *Biochemistry* **1994**, *33*, 7869–7876, doi:10.1021/bi00191a014.
27. Kumagai, I.; Watanabe, K.; Oshima, T. Thermally induced biosynthesis of 2’-O-methylguanosine in tRNA from an extreme thermophile, Thermus thermophilus HB27. *Proceedings of the National Academy of Sciences* **1980**, *77*, 1922–1926, doi:10.1073/pnas.77.4.1922.
28. McCloskey, J.A.; Graham, D.E.; Zhou, S.; Crain, P.F.; Ibba, M.; Konisky, J.; Söll, D.; Olsen, G.J. Post-transcriptional modification in archaeal tRNAs: identities and phylogenetic relations of nucleotides from mesophilic and hyperthermophilic Methanococcales. *Nucleic Acids Res* **2001**, *29*, 4699–4706.
29. Horie, N.; Hara-Yokoyama, M.; Yokoyama, S.; Watanabe, K.; Kuchino, Y.; Nishimura, S.; Miyazawa, T. Two tRNA1Ile species from an extreme thermophile, Thermus thermophilus

HB8: effect of 2-thiolation of ribothymidine on the thermostability of tRNA. *Biochemistry* **1985**, *24,* 5711–5715, doi:10.1021/bi00342a004.
30. Thongdee, N.; Jaroensuk, J.; Atichartpongkul, S.; Chittrakanwong, J.; Chooyoung, K.; Srimahaeak, T.; Chaiyen, P.; Vattanaviboon, P.; Mongkolsuk, S.; Fuangthong, M. TrmB, a tRNA m7G46 methyltransferase, plays a role in hydrogen peroxide resistance and positively modulates the translation of katA and katB mRNAs in Pseudomonas aeruginosa. *Nucleic Acids Res* **2019**, *47*, 9271–9281, doi:10.1093/nar/gkz702.
31. Takano, Y.; Takayanagi, N.; Hori, H.; Ikeuchi, Y.; Suzuki, T.; Kimura, A.; Okuno, T. A gene involved in modifying transfer RNA is required for fungal pathogenicity and stress tolerance of Colletotrichum lagenarium. *Molecular Microbiology* **2006**, *60,* 81–92, doi:10.1111/j.1365-2958.2006.05080.x.
32. Reichle, V.F.; Weber, V.; Kellner, S. NAIL-MS in E. coli Determines the Source and Fate of Methylation in tRNA. *ChemBioChem* **2018**, *19*, 2575–2583, doi:10.1002/cbic.201800525.
33. Durant, P.C.; Davis, D.R. Stabilization of the anticodon stem-loop of tRNALys,3 by an A+-C base-pair and by pseudouridine11Edited by I. Tinoco. *Journal of Molecular Biology* **1999**, *285*, 115–131, doi:10.1006/jmbi.1998.2297.
34. Booth, I.R.; O'Byrne, C.P.; Stansfield, I.; Kinghorn, S.M. Physiological analysis of the role of truB in Escherichia coli: a role for tRNA modification in extreme temperature resistance. *Microbiology* **2002**, *148*, 3511–3520, doi:10.1099/00221287-148-11-3511.
35. Ishida, K.; Kunibayashi, T.; Tomikawa, C.; Ochi, A.; Kanai, T.; Hirata, A.; Iwashita, C.; Hori, H. Pseudouridine at position 55 in tRNA controls the contents of other modified nucleotides for low-temperature adaptation in the extreme-thermophilic eubacterium Thermus thermophilus. *Nucleic Acids Res* **2011**, *39*, 2304–2318, doi:10.1093/nar/gkq1180.
36. Dalluge, J.J.; Hashizume, T.; Sopchik, A.E.; McCloskey, J.A.; Davis, D.R. Conformational flexibility in RNA: the role of dihydrouridine. *Nucleic Acids Res* **1996**, *24*, 1073–1079.
37. Dalluge, J.J.; Hamamoto, T.; Horikoshi, K.; Morita, R.Y.; Stetter, K.O.; McCloskey, J.A. Posttranscriptional modification of tRNA in psychrophilic bacteria. *J Bacteriol* **1997**, *179*, 1918–1923.
38. Chionh, Y.H.; McBee, M.; Babu, I.R.; Hia, F.; Lin, W.; Zhao, W.; Cao, J.; Dziergowska, A.; Malkiewicz, A.; Begley, T.J.; et al. tRNA-mediated codon-biased translation in mycobacterial hypoxic persistence. *Nature Communications* **2016**, *7*, 13302, doi:10.1038/ncomms13302.
39. Müller, M.; Hartmann, M.; Schuster, I.; Bender, S.; Thüring, K.L.; Helm, M.; Katze, J.R.; Nellen, W.; Lyko, F.; Ehrenhofer-Murray, A.E. Dynamic modulation of Dnmt2-dependent tRNA methylation by the micronutrient queuine. *Nucleic Acids Res.* **2015**, *43*, 10952–10962, doi:10.1093/nar/gkv980.
40. Müller, M.; Legrand, C.; Tuorto, F.; Kelly, V.P.; Atlasi, Y.; Lyko, F.; Ehrenhofer-Murray, A.E. Queuine links translational control in eukaryotes to a micronutrient from bacteria. *Nucleic Acids Res* **2019**, *47*, 3711–3727, doi:10.1093/nar/gkz063.
41. Masuda, I.; Matsubara, R.; Christian, T.; Rojas, E.R.; Yadavalli, S.S.; Zhang, L.; Goulian, M.; Foster, L.J.; Huang, K.C.; Hou, Y.-M. tRNA Methylation Is a Global Determinant of Bacterial Multi-drug Resistance. *Cell Systems* **2019**, *8*, 302-314.e8, doi:10.1016/j.cels.2019.03.008.
42. Nair, D.P.; Podgórski, M.; Chatani, S.; Gong, T.; Xi, W.; Fenoli, C.R.; Bowman, C.N. The Thiol-Michael Addition Click Reaction: A Powerful and Widely Used Tool in Materials Chemistry. *Chem. Mater.* **2014**, *26*, 724–744, doi:10.1021/cm402180t.

43. Herzog, V.A.; Reichholf, B.; Neumann, T.; Rescheneder, P.; Bhat, P.; Burkard, T.R.; Wlotzka, W.; von Haeseler, A.; Zuber, J.; Ameres, S.L. Thiol-linked alkylation of RNA to assess expression dynamics. *Nature Methods* **2017**, *14*, 1198–1204, doi:10.1038/nmeth.4435.
44. Schofield, J.A.; Duffy, E.E.; Kiefer, L.; Sullivan, M.C.; Simon, M.D. TimeLapse-seq: adding a temporal dimension to RNA sequencing through nucleoside recoding. *Nature Methods* **2018**, *15*, 221–225, doi:10.1038/nmeth.4582.
45. Yano, M.; Hayatsu, H. Permanganate oxidation of 4-thiouracil derivatives: Isolation and properties of 1-substituted 2-pyrimidone 4-sulfonates. *Biochimica et Biophysica Acta (BBA) - Nucleic Acids and Protein Synthesis* **1970**, *199*, 303–315, doi:10.1016/0005-2787(70)90073-0.
46. Ziff, E.B.; Fresco, J.R. Chemical transformation of 4-thiouracil nucleosides to uracil and cytosine counterparts. *J. Am. Chem. Soc.* **1968**, *90*, 7338–7342, doi:10.1021/ja01028a027.
47. Kramer, G.F.; Baker, J.C.; Ames, B.N. Near-UV stress in Salmonella typhimurium: 4-thiouridine in tRNA, ppGpp, and ApppGpp as components of an adaptive response. *Journal of Bacteriology* **1988**, *170*, 2344–2351, doi:10.1128/JB.170.5.2344-2351.1988.
48. Thiam, K.; Favre, A. Role of the stringent response in the expression and mechanism of near-ultraviolet induced growth delay. *European Journal of Biochemistry* **1984**, *145*, 137–142, doi:10.1111/j.1432-1033.1984.tb08532.x.
49. Favre, A.; Hajnsdorf, E.; Thiam, K.; Caldeira de Araujo, A. Mutagenesis and growth delay induced in Escherichia coli by near-ultraviolet radiations. *Biochimie* **1985**, *67*, 335–342, doi:10.1016/S0300-9084(85)80076-6.
50. Sochacka, E.; Bartos, P.; Kraszewska, K.; Nawrot, B. Desulfuration of 2-thiouridine with hydrogen peroxide in the physiological pH range 6.6–7.6 is pH-dependent and results in two distinct products. *Bioorganic & Medicinal Chemistry Letters* **2013**, *23*, 5803–5805, doi:10.1016/j.bmcl.2013.08.114.
